# Forb composition gradients and intra‐annual variation in a threatened Pacific Northwest Bunchgrass Prairie

**DOI:** 10.1002/ece3.9021

**Published:** 2022-06-22

**Authors:** Joshua P. Averett, Bryan A. Endress

**Affiliations:** ^1^ Eastern Oregon Agricultural Research Center‐ Union Station Oregon State University Union Oregon USA; ^2^ Eastern Oregon Agriculture and Natural Resource Program One University Blvd La Grande Oregon USA

**Keywords:** Bunchgrass Prairie, Grassland forbs, Palouse Prairie, Plant community, Starkey Experimental Forest and Range

## Abstract

Grasslands are among the most threatened and least protected terrestrial biome. Grassland forbs: (1) account for most of the floral diversity; (2) are not well studied because they have been overshadowed by grass‐centered research; and (3) have been a major source for biodiversity loss. The Pacific Northwest Bunchgrass Prairie (PNB) of North America is one of the most endangered grasslands on earth. Knowledge of vegetation community dynamics in the PNB is based primarily on bunchgrasses. There is a paucity of information related to the PNB's diverse native perennial forbs (hereafter “forbs”). Consequently, PNB forb community patterns and dynamics are largely unknown. We describe forb community structure and its relationship to environmental factors and phenology. We sampled 29 plots in the Starkey Experimental Forest and Range, northeastern Oregon, at three different times during 2016 (April; May; July). Nonmetric multidimensional scaling (NMS) indicated that the dominant gradient in forb composition was related (*R* ^2^ = 0.66) to slope and soil P and K, contrasting flat, poorly drained soils (scabflats) at one end with steep, well‐drained soils at the other end. The secondary gradient (*R* ^2^ = 0.16) contrasted deeper, finer textured soils at one end with shallow, rocky soils at the other. Forb richness decreased by ~40% from April to July. NMS and Indicator Species Analysis (ISA) showed that most forbs had affinities toward spring. Ubiquitous forbs (e.g., *Triteleia grandiflora*, *Camassia quamash*) were sparse to absent by July. Contradictory to general descriptions of the PNB, forb cover and richness in drought‐prone sites were comparable to mesic sites when spring data were considered. Our findings suggest that PNB grasslands contain diverse forb communities that are structured primarily by water drainage and soil depth. Conventional sampling concomitant with peak grass biomass is insufficient to characterize PNB forb communities, particularly for scabflats and the most drought‐prone soils.

## Graphical abstract

Native perennial forb communities (an overlooked component in grasslands) in the Pacific Northwest Bunchgrass Prairie (PNB) were structured by water drainage and soil depth. Conventional vegetation sampling is insufficient to characterize perennial forbs in the PNB—threatening conservation efforts in this endangered grassland.

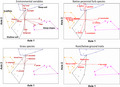

## 1 INTRODUCTION

Grasslands are the most altered and least protected terrestrial biome on earth, are endangered on most continents including North America, and are considered to be among ecosystems with the highest susceptibility for loss of biodiversity over the next century (Bråthen et al., 2021; Henwood, 2010; Sala et al., 2000). Grasslands have already suffered widespread loss of biodiversity, due to anthropogenic activities. Such losses have been particularly severe for forbs (Bråthen et al., 2021). Vegetation descriptions and research in grasslands have focused primarily on the dominant grass species (Gibson, 2009; Siebert & Dreber, 2019; Tisdale, 1982). However, forbs are important for grassland structure and function. Forbs contribute the majority of floral species and functional richness in grasslands (Bond & Parr, 2010; Bråthen et al., 2021; Siebert & Dreber, 2019), influence nutrient and water cycling dynamics (Gould et al., 2016; Tilman et al., 2001), alter soil physical characteristics (Gould et al., 2016), provide important habitat and food resources for vertebrates and invertebrates (Andersen et al., 2018; Haddad et al., 2009; Potts et al., 2009), and are important sources of food and medicine for indigenous people globally (Carbutt et al., 2016; Moerman, 1998; Siebert & Dreber, 2019). Conservation of forbs has been identified as a priority for restoration and protection of the important ecosystem services that grasslands provide (Bråthen et al., 2021; Sheley et al., 2006; Siebert & Dreber, 2019). However, the knowledge needed to support forb conservation and restoration is limited because research focused on the ecology of forb communities in many grasslands is scarce (Bråthen et al., 2021; Pokorny et al., 2004; Siebert & Dreber, 2019) even in some of the world's most threatened grasslands (Endress et al., 2022; Pokorny et al., 2004; Siebert & Dreber, 2019).

Forb species have not been a primary research focus in grasslands; despite their functional importance and high heterogeneity, they have often been treated as secondary components of grasslands and are commonly lumped into overly simplistic categories including all “Forbs” or “other” vegetation (Bråthen et al., 2021; Pokorny et al., 2004; Siebert & Dreber, 2019). Such treatment of grassland forbs has resulted in substantial knowledge gaps regarding forb community ecology and their responses to environmental, abiotic, and disturbance gradients in grasslands (Siebert & Dreber, 2019). One explanation as to why grassland forbs receive comparatively little attention compared to grasses is the ephemeral (short life cycle of above ground biomass) and vernal nature of many forbs resulting in them being unobservable for most of the year and, as a result, underrepresented in grassland sampling that is carried out during summer concomitant with peak production of grasses (Endress et al., 2022; Pokorny et al., 2004, Siebert & Dreber, 2019). Despite, more common spring sampling in European grasslands, vegetation sampling efforts are still likely disproportionately focused on the summer phenological optimum (Vymazalova et al., 2016). Neglecting seasonal differences in vegetation sampling and analyses can have profound effects on syntaxonomic units, and separate sampling and analysis efforts are required to accurately capture and describe spring ephemeral communities (Vymazalova et al., 2016). Seasonal shifts in perennial vegetation composition from spring to summer may be particularly stark for communities that contain a high proportion of geophytes, (Endress et al., 2022; Vymazalova et al., 2016) species with large underground storage organs and short lifespans of above ground biomass. Geophyte rich areas include Mediterranean (Esler et al., 1999), steppe (Daubenmire, 1942), and dry grassland (Vymazalova et al., 2016) ecosystems; one such grassland is the Pacific Northwest Bunchgrass Prairie of North America (PNB; Tisdale, 1982).

The PNB, once covering ~8 million hectares in the intermountain Pacific Northwest is one of the most endangered grasslands in North America with only about 1% remaining in a semi‐natural state (Black et al., 2015; Johnson & Swanson, 2005; Noss et al., 1995; Sampson & Knopf, 1994; Tisdale, 1982). Remnant PNB habitats contain high floral diversity (Darambazar et al., 2007) that is currently threatened by aggressive non‐native annual grass invasion by *Ventenata dubia* and annual *Bromus* species (Endress et al., 2020; Mack, 1981). Current knowledge of vegetation ecology and trends in the PNB are based on the dominant bunchgrass species. Past forb research in the PNB have included descriptions of dominant forb species relationships to bunchgrass compositional gradients (Daubenmire, 1942; Johnson & Simon, 1987; Johnson & Swanson, 2005; Tisdale, 1982), grazing, prescribed fire and non‐native annual grass invasion (Watson et al., 2021), and autecology of specific forbs (U.S. Fish and Wildlife Service, 2007; Taylor et al., 2012; Tubbesing et al., 2014; Weaver, 1915). Yet, there are substantial knowledge gaps related to forb community dynamics and gradients in the PNB, particularly with regard to spring ephemeral species–species whose growth occurs early in the spring and senesce by summer and are abundant in the PNB (Daubenmire, 1942; Tisdale, 1982). Recent research identified that most all vegetation sampling in the PNB is grossly misaligned with forb phenology, that is, sampling takes place after senescence of many forbs (Endress et al., 2022).

The goal of this research was to better understand the ecology of native perennial forbs within the PNB‐a poorly understood component of this highly endangered ecosystem. Specifically, our objectives were to describe forb community structure and its relationship to important environmental factors, and evaluate forb intra‐annual variation in composition to inform optimal timing for forb sampling. Our findings will provide forb community composition and phenology data and inform future conservation, management, monitoring, and research efforts related to grassland forb community ecology.

## 2 STUDY AREA

Research occurred in the grasslands of Starkey Experimental Forest and Range (SEFR) in northeastern Oregon (45°12 N, 118°3 W). Elevations range between 1120 and 1500 m, precipitation averages 510 mm annually with a pronounced drought in summer; average annual temperatures range from −4°C in winter to 18°C in summer (Rowland et al., 1997). Bunchgrass composition varies along soil depth and water availability gradients from *Poa secunda* and *Pseudoroegneria spicata* dominance on the most droughty soils (e.g., well‐drained soils and south aspects) to *F. idahoensis* and *Koeleria macrantha* in the most mesic grassland sites (e.g., fine‐textured soils and north aspects). *Poa secunda* and *Danthonia unispicata* are dominant on the shallowest soils (Johnson & Swanson, 2005).

## 3 METHODS

We used a stratified random design to sample vegetation composition from 29 (154 m^2^) plots in PNB habitat within the SEFR. Areas within the Main and Campbell study areas (8385 ha total; Rowland et al., 1997) were stratified by percent tree cover (2011 National Land Cover Dataset; Jin et al., 2013), then 30 plots were randomly located within areas with ≤5% tree cover (grassland plots). One plot was located within an open forest when visited, and therefore, excluded, resulting in a total of 29 plots. We sampled each plot at three different times during the growing season in 2016: April (April 18th–May 2nd); May (May 23rd–June 1st); and July (July 11th–18th). The first two sample periods coincided with growth of spring ephemerals. The third was within the traditional vegetation sampling window for PNB (early summer; Endress et al., 2022).

### 3.1 Vegetation sampling

One circular plot (radius = 7 m) was established at each sampling site and 12 quadrats (1 m^2^) were systematically located within each plot (Appendix S1: Figure A1). Two transect lines were laid out perpendicular to each other and intersecting at the center of the plot, resulting in one line running 14 m in length from north (0 m) to south (14 m), and the other 14 m in length from west (0 m) to east (14 m). Four quadrats were centered at 0.5 m, 4 m, 10 m, and 13.5 m along each of the two transects for a total of eight quadrats along the north/south and west/east lines. Four additional quadrats were located 4 m from the plot center along each of the NE, SE, SW, and NW cardinal directions for a total of 12 quadrats per plot (Appendix S1: Figure A1).

Within each quadrat, presence/absence was recorded and canopy cover was estimated for all forb species during the early (April and May) sampling periods, and all vascular plant species during the late (July) sampling period. Canopy cover was classified into one of eight cover categories (≤1%; >1–5%; >5–25%; >25–50%; >50–75%; >75–95%; >95–99%; >99–100%). Cover classes, yield statistical results similar to unclassed data, do not attempt to achieve more accuracy than is realistic, they effectively represent biomass measurements, and have been used to successfully detect community change over time (McCune & Grace, 2002). Plot level abundance for each species was calculated as the frequency of quadrats occupied per plot. Plot‐level cover was calculated as the average arithmetic midpoint of the cover classes. Graminoids (grasses, sedges, and rushes) were not identified to species during April and May due to their early phenological development. Plot‐level cover of the soil surface, that is, litter, rock, biotic crust (moss or lichen), and bare ground along with cover of total vegetation and each functional group (i.e., perennial forbs, annual forbs, perennial graminoids, annual graminoids, and shrubs) were estimated the same as species cover.

### 3.2 Environmental variables

A tile probe was used to measure depth to soil restrictive layer (average of nine samples per plot) within 80 cm of the mineral soil surface during April at the center of the plot and at 1.75 m and 12.25 m along the North/South and West/East lines as well as at 6.5 m from the plot center along each of the NE, SE, SW, and NW lines. Nine soil cores (≤25 cm deep depending on soil depth) were collected offset (0.25 m toward the plot center) from the tile probe measurement locations (Appendix S1: Figure A1). The nine soil cores were then mixed for each plot, ground, and dried at 60°C for 48 h. Soil chemical and textural analyses were performed at AgSource Laboratory (Umatilla, OR, USA) for pH, cation exchange capacity (CEC), organic matter (%), phosphorous (P; Olsen), potassium (K; ammonium acetate), magnesium (Mg; ammonium acetate), calcium (Ca; ammonium acetate), sodium (Na; ammonium acetate), and percent sand, silt, and clay.

Elevation, slope, and aspect were extracted from 30‐m resolution digital elevation models (U.S. Geological Survey 2006) using ArcGIS 9.1. We transformed aspect by folding the aspect about the NE–SW lines (McCune & Keon, 2002) to align with an expected heat load gradient (SW = maximum heat load and NE = minimum heat load).

### 3.3 Statistical Analysis

Plot level species abundance (frequency; percent of quadrats occupied per plot) was calculated for each sampling period (April, May, July) separately and also for the combination of all sampling periods (peak abundance). Peak abundance was calculated for each species in a plot as the maximum abundance for that species in that plot over the three sampling periods. Total species richness and diversity were calculated for both the July and combined (all time periods) datasets. Because graminoids were not identified to species during April and May, only forb richness and diversity could be calculated for the earlier time periods.

#### 3.3.1 Community gradients

Nonmetric Multidimensional Scaling (NMS; McCune & Mefford, 2015) using a Sørensen distance measure, the “slow and thorough” autopilot setting and Kruskal's strategy 2 for penalization for ties in the distance matrix was used to extract the dominant native perennial forb community composition gradients. NMS was run with a random starting configuration and maximum of 500 iterations. Rare species (occurring in less than two out of the 29 plots) were removed from the species matrix prior to ordination to reduce noise and enhance any signal related to dominant species compositional relationships with environmental variables (McCune & Grace, 2002). Multivariate analyses were performed on the final species matrix of 29 plots by 53 forb species (Table 1); rows were plots, columns were species, and each cell was the “peak” abundance for a particular species in that plot. Species abundances were transformed using a generalized log transformation to give subdominant species more weight in the ordination structure while maintaining monotonicity with the raw data (McCune & Grace, 2002). By giving more weight to subdominant species, this allows more information regarding those species to be used in defining the ordination space instead of relying on only a few very dominant species to drive definition of the ordination space. Whereas extremely rare species add tremendously to the bulk noise and add little information related to community dissimilarity, subdominant species are common enough that they add little noise but contain a lot of information (McCune & Grace, 2002). Beta diversity (*B*_*w*_), the average compositional difference among plots (McCune & Grace, 2002) was 1.6 after deletion of rare species and log transformation.

**TABLE 1:** Native perennial forb mean abundance (mean quadrat frequency per plot) by sampling period and linear correlation coefficients (*r*) with NMS ordination axes

Species	Abundance (% of quadrats/plot)			Axis 1	Axis 2	Group Tendency	IV	*p*‐value
	April	May	July					
*Achillea millefolium*	33.62	37.64	34.20	0.63	0.14	Early	37.0	.903
***Agoseris grandiflora***	**8.33**	**12.36**	**0.29**	**0.09**	**−0.11**	**Early**	**53.7**	**<.001**
***Allium acuminatum***	**4.60**	**12.36**	**0.29**	**0.03**	**−0.68**	**Early**	**23.3**	**.016**
*Allium fibrillum*	16.09	16.95	9.20	−0.04	−0.12	Early	16.7	.418
***Allium tolmiei***	**18.97**	**18.10**	**3.74**	**−0.41**	**−0.67**	**Early**	**24.4**	**.067**
*Antennaria* spp.	9.48	10.34	7.47	−0.29	−0.32	Early	34.4	.527
*Apocynum androsaemifolium*	1.44	2.01	2.59	0.39	0.03	NA	NA	NA
*Arnica sororia*	10.63	10.06	4.02	0.08	0.61	Early	17.4	.208
*Astragalus reventus*	8.91	8.91	6.61	0.66	−0.30	NA	NA	NA
***Balsamorhiza incana***	**10.06**	**0.86**	**0.57**	**−0.44**	**−0.32**	**Early**	**17.2**	**.059**
*Balsamorhiza serrata*	6.03	16.09	16.38	−0.58	−0.49	Late	26.8	.210
Besseya rubra*	0.57	0.57	0.29	NA	NA	NA	NA	NA
*Calochortus elegans*	1.15	1.44	0.00	−0.14	0.14	NA	NA	NA
Calochortus eurycarpus*	0.00	0.00	0.29	NA	NA	NA	NA	NA
***Camassia quamash***	**60.06**	**58.62**	**28.16**	**−0.83**	**0.10**	**Early**	**51.4**	**.009**
Camissonia subacaulis*	0.00	0.29	0.00	NA	NA	NA	NA	NA
***Castilleja* spp.**	**10.06**	**7.47**	**22.13**	**−0.56**	**0.41**	**Late**	**34.6**	**.013**
***Claytonia lanceolata***	**10.06**	**0.29**	**0.00**	**−0.00**	**0.25**	**Early**	**17.2**	**.043**
*Crepis acuminata*	0.29	1.72	0.00	0.10	−0.02	NA	NA	NA
***Crepis* spp.**	**18.68**	**14.66**	**2.30**	**0.20**	**−0.20**	**Early**	**43.9**	**<.001**
***Delphinium bicolor***	**43.97**	**29.89**	**11.78**	**0.15**	**0.13**	**Early**	**56.2**	**<.001**
*Dodecatheon pulcherimum*	3.16	2.30	0.29	−0.19	0.26	Early	12.5	.167
*Eriogonum heracleoides*	13.79	14.37	13.51	0.70	0.05	Early	20.2	.909
*Erigeron linearis*	0.86	1.15	1.44	0.25	−0.06	NA	NA	NA
Eriophyllum lanatum*	0.86	2.01	1.44	NA	NA	NA	NA	NA
***Fritillaria pudica***	**28.45**	**6.61**	**0.29**	0.14	0.31	**Early**	**50.9**	**<.001**
*Galium boreale*	0.00	0.00	2.30	0.59	−0.19	NA	NA	NA
Geum triflorum*	0.86	0.86	0.57	**NA**	**NA**	NA	NA	NA
*Grindelia nana*	8.33	12.64	12.93	−0.10	−0.15	Late	22.8	.683
***Hesperochiron pumilus***	**36.49**	**25.00**	**1.44**	−0.55	0.36	**Early**	**56.0**	**<.001**
Hieracium cynoglossoides*	0.29	0.00	0.29	NA	NA	NA	NA	NA
Hydrophyllum capitatum*	0.29	0.00	0.00	NA	NA	NA	NA	NA
Ipomopsis aggregate*	0.00	0.00	0.57	NA	NA	NA	NA	NA
***Lithophragma glabrum***	**83.62**	**15.80**	**1.15**	−0.57	−0.10	**Early**	**80.9**	**<.001**
***Lithophragma parviflorum***	**12.07**	**8.62**	**0.86**	0.61	0.42	**Early**	**30.2**	**.008**
*Lomatium ambiguum*	7.76	1.15	0.29	**0.29**	**0.41**	Early	9.7	.192
*Lomatium bicolor*	60.34	54.31	37.07	**−0.82**	**−0.03**	Early	44.0	.168
***Lomatium cous***	**20.98**	**20.40**	**3.16**	0.26	−0.51	**Early**	**22.4**	**.076**
*Lomatium macrocarpum*	7.18	7.47	3.45	0.59	−0.33	Early	11.7	.442
*Lomatium nudicale*	2.01	2.30	1.15	**−0.22**	**−0.19**	NA	NA	NA
Lomatium triternatum*	0.29	0.00	0.00	NA	NA	NA	NA	NA
*Lupinus* spp.	6.90	4.89	4.60	0.08	0.14	NA	NA	NA
*Lupinus sulphureus*	0.00	3.16	0.00	0.36	0.00	NA	NA	NA
***Mertensia longiflora***	**6.32**	**0.86**	**0.00**	**−0.04**	**0.13**	**Early**	**17.2**	**.045**
*Microseris nutans*	14.08	11.21	9.48	−0.25	−0.06	Early	19.7	.900
*Nothocalais troximoides*	0.29	1.15	0.00	−0.26	0.09	NA	NA	NA
*Olsynium douglasii*	64.66	61.78	48.85	−0.78	0.09	Early	51.5	.110
***Orogenia linearifolia***	**30.46**	**0.86**	**0.00**	**0.13**	**0.11**	**Early**	**34.5**	**.002**
*Perideridia gairdneri*	7.47	14.66	15.52	0.05	0.76	Late	22.1	.179
*Potentilla glandulosa*	0.57	0.29	0.57	−0.04	0.40	NA	NA	NA
*Potentilla gracilis*	2.01	2.59	2.01	−0.07	0.48	NA	NA	NA
***Ranunculus glaberrimus***	**42.82**	**1.15**	**0.00**	**−0.16**	**0.13**	**Early**	**41.4**	**<.001**
***Saxifraga nidifica***	**23.85**	**10.92**	**5.46**	**−0.22**	**0.77**	**Early**	**31.2**	**.034**
*Scutellaria angustifolia*	2.59	3.16	2.59	NA	NA	NA	NA	NA
*Sedum stenopetalum*	13.51	14.37	12.93	−0.42	−0.54	Early	14.3	.919
Selaginella wallacei*	0.00	0.00	2.87	NA	NA	NA	NA	NA
*Sidalcea oregana*	4.60	5.75	5.46	0.13	0.65	Early	10.9	.965
*Trifolium eriocephalum*	3.74	6.90	0.00	−0.06	0.42	NA	NA	NA
*Trifolium macrocephalum*	9.48	10.06	6.32	−0.46	−0.10	Early	12.6	.499
*Trifolium plumosum*	0.29	0.00	0.29	−0.14	0.01	NA	NA	NA
***Triteleia grandiflora***	**77.30**	**46.84**	**1.15**	**0.45**	**0.08**	**Early**	**88.0**	**<.001**
*Viola adunca*	0.86	0.29	0.00	−0.08	0.37	NA	NA	NA
Wyethia amplexicaulis*	0.57	0.29	0.57	NA	NA	NA	NA	NA
*Zigadenus venenosus*	1.72	1.44	1.15	0.31	−0.02	Early	8.0	.710

*Note*: The species matrix for this NMS ordination were native perennial forb abundances for the three time periods, that is, 64 species (columns) by 87 sample units—29 plots for each of the three time periods (rows). Species indicator values (IV) are reported with sampling period tendency (early, April and May; late, July) and significance of IVs. Species IVs with *p*‐values ≤.05 are shown in bold. Asterisks indicate rare species (occurred in <2 plots) that were removed before creation of the NMS ordination space.

**TABLE 2:** Means and standard deviations (represented parenthetically) for environmental variables stratified by vegetation group

Group	Elevation (m)	Slope (%)	Soil depth (cm)	K (ppm)	P (ppm)	Soil pH	Clay (%)	Rock (%)	Bare ground (%)
1	1446 (18)	4.2 (2.5)	14.4 (2.8)	168 (26)	10.0 (2.0)	5.8 (0.2)	8.2 (1.8)	4.8 (2.7)	9.7 (12.6)
2	1334 (114)	10.9 (5.7)	10.1 (3.1)	178 (29)	10.8 (1.6)	5.9 (0.1)	7.4 (2.4)	9.5 (7.1)	22.5 (15.6)
3	1233 (95)	5.7 (2.6)	26.6 (10.5)	219 (45)	13.4 (3.5)	5.9 (0.1)	9.4 (1.4)	2.1 (3.1)	7.3 (5.3)
4	1266.2 (58)	29.6 (15.6)	19.1 (2.4)	294 (59)	21.3 (4.8)	6.2 (0.2)	8.2 (2.0)	7.9 (5.7)	26.0 (4.4)

*Note*: K, P, and clay (%) were derived from soil samples. Rock (%) and Bare ground (%) are plot level averages of ocular estimates of percent cover of each variable in quadrats.

A forb trait matrix was constructed to relate forb root characteristics to community gradients and environmental variables. In order to create the trait matrix, perennial forb functional group classification defined in (Endress et al., 2022) (categorized by root and underground storage morphology into one of four categories) were used: (1) geophyte, species with bulbs, tubers, corms, cormlets, or underground bulblets; (2) fibrous‐rooted forbs; (3) tap‐rooted forbs; and (4) rhizomatous forbs. For each species, a “one” was entered into the matrix for the category they matched, and a “zero” for any trait that did not match. If a species had more than one dominant root trait (e.g., tap‐rooted and strongly rhizomatous) then that species received a “one” for each of the two categories. The resulting matrix was then transposed and multiplied by the species abundance matrix (McCune & Grace, 2002) to obtain a trait abundance matrix where each cell represented the abundance of a particular trait in that plot. The final trait matrix for analyses was constructed by performing a general relativization (McCune & Grace, 2002) by row totals to the trait abundance matrix to focus on the relative abundance of each perennial forb trait (life‐form) to the total perennial forb abundance in each plot. Joint plots were created to evaluate linear relationships between species abundance, trait (root/underground storage morphology) relative abundance, and environmental variables with the dominant forb composition gradients (McCune & Grace, 2002). We chose to focus on root morphology because root traits are strongly influenced by growing season drought conditions (Comas et al., 2013) the dominant driver of grassland structure in dry grasslands (Burke et al., 1998) including bunchgrass composition in the PNB (Daubenmire, 1942; Johnson & Swanson, 2005). Examining relationships between root traits and community/environmental variation provides a starting point to understand general patterns of forb functional group variation in the PNB. We acknowledge that other traits including leaf characteristics are important for explaining variation in plant adaptations to different environmental conditions. Future research will be needed to understand how a whole suite of forb traits relate to community and environmental factors in the PNB and similar grasslands.

Because most previous vegetation research in the PNB was based on bunchgrass composition, bunchgrass species abundances were related to the ordination space using joint plots. We also tested the null hypothesis of no correspondence between native perennial forb distribution and perennial bunchgrass distribution using a Mantel test (McCune & Grace, 2002).

Cluster analysis with a Euclidean distance measure and Wards method for clustering was used in PC‐ORD 7.0 to identify natural groupings of species in our dataset (McCune & Mefford, 2015). The optimal number of groups were chosen using indicator species analysis (ISA; McCune & Grace, 2002). The number of groups with the highest number of significant indicator species and/or the lowest average p‐value can be used to select the optimal number of groups for pruning cluster diagrams (Dufrêne & Legendre, 1997; McCune & Grace, 2002); both criteria were met with four groups and resulted in natural grouping (presence of long stems indicating increased homogeneity within groups) and ~36% of information remaining in the distance matrix (Appendix S1: Table 2, Tables A2 and S2).

Nonparametric multiplicative regression (NPMR; HyperNiche 2.30; McCune & Mefford, 2009), a multiplicative kernel smoother which automatically models interactions among predictors, and has a built‐in over‐fitting protection (McCune, 2006) was used to evaluate species’ nonlinear responses to the ordination space as well as to identify important predictors of forb species abundance. Model fit was evaluated using Cross‐validated *R* ^2^ (*xR* ^2^) which differs from *R* ^2^ because it is based on the exclusion of each data point from the estimate of the response at that point (McCune, 2006). Models were considered up to a maximum of three predictors. A predictor was added to the model if it increased the *xR* ^2^ by at least 0.03 (Averett et al., 2016). Predictor importance was evaluated by its sensitivity. Sensitivity is the ratio of the relative mean difference in the response to the relative mean difference in the predictor, that is, a sensitivity of 1.0 indicates that a 5% change in the predictor would elicit a 5% change in the response (McCune, 2006).

#### 3.3.2 Intra‐annual variation

Blocked Multi Response Permutation Procedure (MRBP; McCune & Mefford, 2015) was used to test the null hypothesis of no change in native perennial forb composition between sampling periods (after deletion of rare species) after accounting for block differences. We used a Euclidean distance measure and aligned the medians to zero for all blocks (McCune & Grace, 2002). Pairwise comparisons were made to test for differences in species composition between all pairs of sampling periods, and the Bonferroni procedure was used to adjust *p*‐values for multiple comparisons.

NMS was used to extract the dominant gradients in native perennial forb composition across all three sampling periods using the same criteria explained above. The final species matrix for this analysis consisted of 87 plots (29 for each sampling period) by 53 forb species. Plots were grouped by sample period to explore any relationship between sample timing and the ordination space. A joint plot was used to explore linear relationships between sample timing and species abundances and environmental variables. Indicator Species Analysis (ISA; McCune & Mefford, 2015) was used to identify forb affinities toward either the early (April or May) or late (July) sample periods. ISA uses both relative abundance and relative frequency information to generate an Indicator Value (IV) for each species; an IV of zero indicates that a species never occurs in a particular group, whereas an IV of 100 is a measure of perfect indication (the species only and always occurs in the group of interest). Significance of IV's was determined by comparing observed values with results from 10,000 randomizations of the data.

## 4 RESULTS

### 4.1 Richness and dominance

**FIGURE 1:**
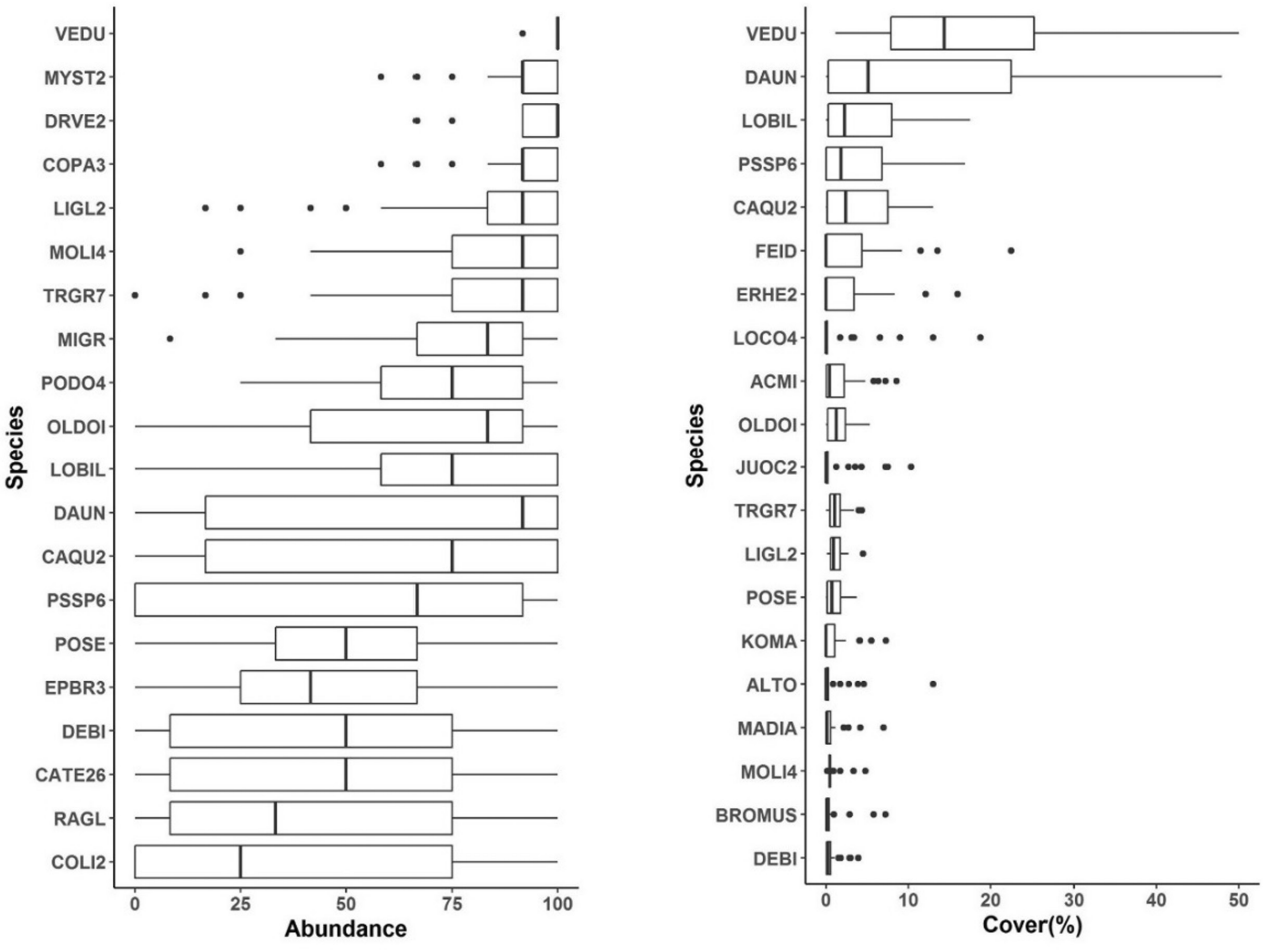
Species abundance (% of quadrats occupied per plot; left panel), and canopy cover (right panel) ordered by dominance (top to bottom) for the twenty most dominant species (over three sampling periods; April, May, & July) in each abundance category across 29 randomly located plots within bunchgrass habitats in the Starkey Experimental Forest and Range. ACMI (*Achillea millefolium*); ALTO (*Allium tolmiei*); BROMUS (annual *Bromus* species); CAQU2 (*Camassia quamash*); CATE26 (*Castilleja tenuis*); COLI2 (*Collomia linearis*); COPA3 (*Collinsia parviflora*); DAUN (*Danthonia unispicata*), DEBI (*Delphinium bicolor*); DRVE2 (*Draba verna*); EPBR3 (*Epilobium brachycarpum*); ERHE2 (*Eriogonum heracleoides*); FEID (*Festuca idahoensis*); JUOC2 (*Juncus occidentalis*); KOMA (*Koeleria macrantha*); LIGL2 (*Lithophragma glabrum*); LOBIL (*Lomatium bicolor*); LOCO4 (*Lomatium cous*); MADIA (*Madia* spp.); MIGR (*Microsteris gracilis*); MOLI4 (*Montia linearis*); MYST2 (*Myosotis stricta*); OLDOI (*Olsynium douglasii*); PODO4 (*Polygonum douglasii*); POSE (*Poa secunda*); PSSP6 (*Pseudoroegneria spicata*); RAGL (*Ranunculus glaberrimus*); TRGR7 (*Triteleia grandiflora*)

A total of 119 vascular plant species (67 perennial forbs, 32 annual forbs, 14 perennial graminoids, 5 annual grasses, and 1 shrub species) including 102 (86% of species) native and 17 (14% of species) non‐native species were observed over the three sampling periods. Forb (annual and perennial) richness made up 83% (99 out of 119 species) of total richness. We identified 64 native perennial forbs (making up ~54% of vascular plant richness; Table 1). Mean plot‐level species richness across the three sampling periods was 45.6 compared to 28.1 in July (two sample *t*‐test, *t* = −11.33, p‐value <0.001, estimated difference between the total plot‐level richness [April, May, and July] and July = 17.5, 95% CI = 14.4 to 20.6). The five most abundant species included four annuals, the top three were non‐native to the region, *V. dubia* (introduced annual grass; IAG), *Myosotis stricta* (introduced annual forb; IAF), *Draba verna* (IAF), *Collinsia parviflora* (native annual forb; NAF), followed by *Lithophragma glabrum* (native perennial forb; NPF; Figure 1). Species with the highest mean cover estimates were *V. dubia* (IAG), *Danthonia unispicata* (native perennial grass; NPG), *Lomatium bicolor* (NPF), *Pseudoroegneria spicata* (NPG), and *Camassia quamash* (NPF; Figure 1).

### 4.2 Community gradients

#### 4.2.1 Nonmetric multidimensional scaling ordination

**FIGURE 2:**
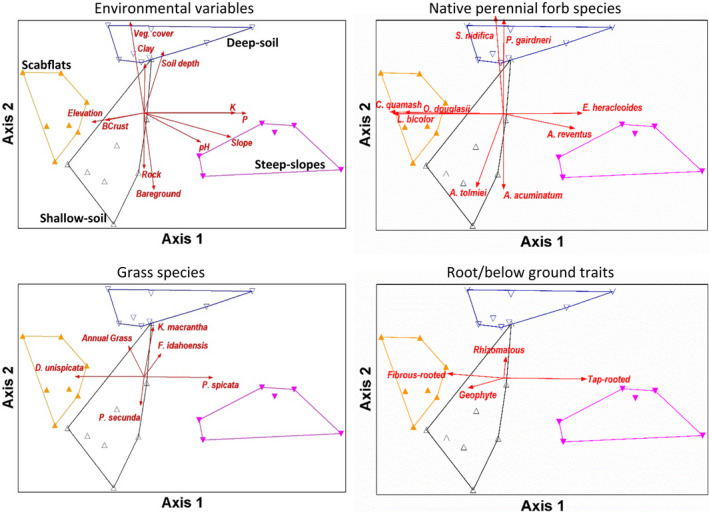
NMS ordination of plots (triangles) in native perennial forb species space. Axis 1 (explaining 66% of variation in the distance matrix) and axis 2 (explaining 16%) show the dominant community composition gradients for this data set. Vectors show direction and strength (length of vectors) of linear correlations for (top‐left) environmental (top‐right) native perennial forb species abundance, (bottom‐left) grass species abundance, and (bottom‐right) forb trait relative abundance with the ordination space (Axes 1 & 2). Convex hulls show vegetation groups derived from pruning the cluster dendrogram at four groups

The optimal solution was a two‐dimensional ordination (Figure 2; final stress = 13.6, randomization test *p*‐value = 0.004) that explained 81.9% of variation in the distance matrix. Groups identified with cluster analysis (Figure 2; Appendx S1: Table 2, Tables A2 and S2) separated clearly in ordination space (Figure 2). Axis 1 explained most of the variation (66%) in the distance matrix and was most strongly related to soil P (*r* = 0.82), K (0.77), slope (0.75), soil pH (0.61), and elevation (−0.61). Along Axis 1, slope and soil P, K, and pH all increased while elevation (*r* = −0.61) decreased from left to right (Figure 2). Species with the strongest negative relationships to Axis 1 included *C. quamash* (*r* = −0.83), *L. bicolor* (−0.82), *Olsynium douglasii* (−0.78), and *L. glabrum* (−0.57; Figure 2; Table 1). Species with the strongest positive relationships to Axis 1 were *Eriogonum heracleoides* (0.70), *Astragalus reventus* (0.66), *Achillea millefolium* (0.63), and *Lithophragma parviflorum* (0.61; Figure 2; Table 1). Relative abundances of fibrous‐rooted perennials (*r* = −0.52) and geophytes (−0.41) were negatively related to Axis 1 (Figure 2).

Axis 2 explained 15.9% of variation in the distance matrix and was most positively related to total vegetation cover (*r* = 0.77), soil depth (0.63), and percent clay (0.57; Figure 2). Soil depth and texture characteristics create a water availability gradient, that is, deeper, finer‐textured soils high along Axis 2 indicate the highest water availability in our study area and shallow, coarser‐textured soils low along Axis 2 correspond to the lowest water availability. Environmental factors with the strongest negative relationships with Axis 2 were percent cover of bare ground (−0.71) and percent cover of rock (−0.61; Figure 2). *Saxifraga nidifica* (0.77) and *Perideridia gairdneri* (0.76) had the strongest positive relationships with Axis 2 (Figure 2). Species with the strongest negative relationships to Axis 2 were *Allium acuminatum* (−0.68), *Allium tolmiei* (−0.67), *Sedum stenopetalum* (−0.54), and *Balsamorhiza serrata* (−0.49; Figure 2; Table 1). Rhizomatous forbs were positively correlated (*r* = 0.32) with Axis 2 and geophyte relative abundance (−0.22) had a slight negative relationship with Axis 2 (Figure 2).

NMS axes were also associated with changes in bunchgrass composition (Figure 2). Along Axis 1, there was a clear gradient of decreasing *D. unispicata* (*r* = −0.85) and increasing *P. spicata* (0.68) from left to right (Figure 2). Along Axis 2, *P. secunda* (*r* = −0.55) cover decreased as *K. macrantha* (0.59) cover increased from low to high (Figure 2). Non‐native annual grass cover was highest in the top left corner of the ordination space and *F. idahoensis* (native bunchgrass) cover was greatest in the top right corner of the ordination space (Figure 2). Forb community dissimilarity had a moderate association with native perennial bunchgrass community dissimilarity (Mantel test, *r* = 0.560, *p* < .001); that is, the distribution of native bunchgrasses were moderately correlated with the distribution of native perennial forbs suggesting that grass and forb species may be, at least partly, filtered by the same environmental gradients.

#### 4.2.2 Forb relationships to ordination axes and environmental predictors

NPMR models revealed moderate to strong relationships between the ordination space and the abundance of about a third of the common forbs including *C. quamash* (*xR* ^2^ = 0.82*)*, *A. tolmiei* (0.78), *P. gairdneri* (0.69), and *S. nidifica* (0.69; Table 3). Other forbs, for example, *Grindelia nana* (−0.07), *Fritillaria pudica* (0.19), and *Trifolium macrocephalum* (0.13) were only weakly related to the ordination axes (Table 3). Two or three predictor models, using environmental variables as predictors, explained more variation in the abundance of some forbs (that were weakly related to NMS axes) compared to the ordination axes (Table 3). About 75% of variation in *G. nana* abundance was explained by CEC (sensitivity = 0.43), Elevation (0.31), and K (0.09; Table 3). Approximately 42% and 40% of variation in *F. pudica* and *Tri. macrocephalum* abundance was explained by Mg (sensitivity = 0.49), K (0.35), and C (0.21) and Mg (0.35), clay (0.23), and Na (0.22), respectively (Table 3). NMS axes and environmental variables explained little variation (<22%) for some of the most ubiquitous forbs (e.g., *L. glabrum*, *Delphinium bicolor*, *Ranunculus glaberrimus*) in our study area (Table 3).

**TABLE 3:** Summary of NPMR models for native perennial forb abundance against NMS axes and best environmental predictors (far right column) with associated *xR* ^2^ and predictor sensitivities

Species	NMS Axes Models		Axis 1		Axis 2		Best Environmental Predictors (sensitivity)
	xR^2^	Neighb	Tolerance	Sensitivity	Tolerance	Sensitivity	
*Achillea millefolium*	**0.530**	2.3	0.151	1.178	0.565	0.182	*xR* ^2^ = **0.72**: Clay (0.64), Aspect (0.16), Elevation (0.14)
*Allium acuminatum*	**0.593**	2.6	0.454	0.199	0.188	0.658	*xR* ^2^ = **0.52**: K (0.41), Aspect (0.26), Elevation (0.15)
*Allium fibrillum*	0.172	6.9	0.302	1.030	1.50	0.029	*xR* ^2^ = 0.37: Slope (1.2), Elevation (0.26)
*Allium tolmiei*	**0.781**	3.2	0.605	0.265	0.188	0.816	*xR* ^2^ = **0.66**: Ca(1.4), Mg(0.32)
*Antennaria* spp.	0.422	1.5	0.454	0.302	0.094	0.910	*xR* ^2^ = 0.22: P(1.3), pH(0.18), Clay(0.17)
*Arnica sororia*	0.298	5.8	0.605	0.186	0.377	0.3945	*xR* ^2^ = 0.46: Soil depth(0.76), Aspect(0.03)
*Balsamorhiza incana*	0.112	9.4	0.605	0.372	0.754	0.127	*xR* ^2^ = 0.15: K(1.2), P(0.16), Elevation(0.12)
*Balsamorhiza serrata*	**0.535**	3.3	0.302	0.743	0.829	0.305	*xR* ^2^ = **0.53**, Soil depth(0.53), Aspect(0.22), Elevation(0.09)
*Camassia quamash*	**0.819**	2.3	0.151	0.572	0.565	0.090	*xR* ^2^ = **0.69**: Aspect(0.34), P(0.24), Ca(0.22)
*Castilleja* spp.	0.371	1.5	0.454	0.458	0.094	1.479	*xR* ^2^ = 0.46: Slope(1.8), Aspect(0.08)
*Claytonia lanceolata*	0.124	1.5	0.454	0.278	0.094	1.262	*xR* ^2^ = **0.52**: CEC(1.8), Slope(0.24)
*Crepis* spp.	−0.071	22.2	2.120	0.035	1.508	0.026	*xR* ^2^ = 0.20: Mg(0.80), Aspect(0.58)
*Delphinium bicolor*	−0.084	22.8	1.873	0.018	0.829	0.004	*xR* ^2^ = 0.02: Slope(0.37), Soil depth(0.36), P (0.26)
*Dodecatheon pulcherimum*	−0.039	9.4	1.873	0.109	0.829	0.086	*xR* ^2^ = 0.26: Na(0.27), K(0.15)
*Eriogonum heracleoides*	0.385	6.9	0.302	0.789	1.508	0.018	*xR* ^2^ = **0.58**: Na(0.41), Elevation(0.39), Mg(0.07)
*Fritillaria pudica*	0.190	1.9	0.302	0.780	0.188	0.652	*xR* ^2^ = 0.42: Mg(0.49), K(0.35), Ca(0.21)
*Grindelia nana*	−0.073	14.3	0.757	0.143	1.508	0.030	*xR* ^2^ = **0.75**: CEC(0.43), Elevation(0.31), K(0.09)
*Hesperochiron pumilus*	0.425	1.9	0.302	0.738	0.188	0.617	*xR* ^2^ = 0.36: Na(0.53), Clay(0.41), (K(0.05)
*Lithophragma glabrum*	0.215	9.8	0.454	0.334	1.508	0.023	*xR* ^2^ = 0.21: Na(0.39), Elevation(0.02)
*Lithophragma parviflorum*	0.451	4.6	0.454	0.564	0.377	0.335	*xR* ^2^ = **0.50**: P(1.4), Aspect(0.10), Elevation(0.07)
*Lomatium ambiguum*	0.073	2.6	0.302	0.311	0.829	0.162	*xR* ^2^ = 0.34: Aspect(1.4), pH(0.20), Elevation(0.03)
*Lomatium bicolor*	**0.674**	6.9	0.302	0.817	1.508	0.009	*xR* ^2^ = **0.78**: Na(1.1), Elevation(0.05), Aspect(0.03)
*Lomatium cous*	0.451	2.6	0.302	0.855	0.282	0.404	*xR* ^2^ = 0.49: Slope(2.1), CEC(0.12), Na(0.08)
*Lomatium macrocarpum*	0.293	7.8	0.605	0.371	0.565	0.165	*xR* ^2^ = **0.65**: P(0.89), Aspect(0.08)
*Mertensia longiflora*	−0.040	6.0	2.423	0.010	0.788	0.455	*xR* ^2^ = 0.27: Soil depth(0.46), CEC(0.19), Aspect(0.06)
*Microseris nutans*	0.033	6.9	0.302	0.993	1.508	0.023	*xR* ^2^ = 0.32: Aspect(1.8), pH(0.32), Ca(0.13)
*Olsynium douglasii*	0.488	9.8	0.454	0.468	1.508	0.007	*xR* ^2^ = **0.74**: Na(0.23), Soil depth(0.22), Mg(0.10)
*Orogenia linearifolia*	0.179	6.9	0.302	1.183	1.508	0.020	*xR* ^2^ = 0.28: Slope(0.91), Mg(0.72)
*Perideridia gairdneri*	**0.687**	4.5	0.605	0.211	0.282	0.585	*xR* ^2^ = **0.61**: Aspect(1.05), Soil depth(0.35), Elevation(0.09)
*Ranunculus glaberrimus*	−0.043	12.3	0.605	0.321	1.508	0.012	*xR* ^2^ = 0.17: P(0.54), Ca(0.34), Elevation(0.09)
*Saxifraga nidifica*	**0.692**	6.0	2.423	0.021	0.188	0.745	*xR* ^2^ = **0.62**: Soil depth(0.77), Na(0.42), Aspect(0.06)
*Sedum stenopetalum*	0.403	4.6	0.454	0.344	0.377	0.401	*xR* ^2^ = **0.64**: Ca(0.45), Elevation(0.29), Clay(0.07)
*Sidalcea oregana*	**0.628**	2.4	0.908	0.066	0.094	0.847	*xR* ^2^ = 0.43: Soil depth(0.24), Slope(0.19), CEC(0.18)
*Triteleia grandiflora*	0.447	1.5	0.454	0.271	0.094	0.565	*xR* ^2^ = 0.19: P(0.36), Elevation(0.10), Mg(0.07)
*Trifolium macrocephalum*	0.125	6.9	0.302	0.663	1.508	0.013	*xR* ^2^ = 0.41: Mg(0.35), Clay(0.23), Na(0.22)
*Zigadenus venenosus*	−0.051	17.4	1.060	0.120	0.829	0.006	*xR* ^2^ = 0.40: Slope(0.21), pH(0.08)

*Note*: *xR* ^2^ ≥ 0.5 are depicted in bold font. “Neighb” is the average neighborhood size (amount of data used for estimates at each point); Tolerance is how broadly, expressed in range of the predictor, estimates are based on surrounding sample data (McCune & Grace, 2002).

### 4.3 Intra‐annual variation

**FIGURE 3:**
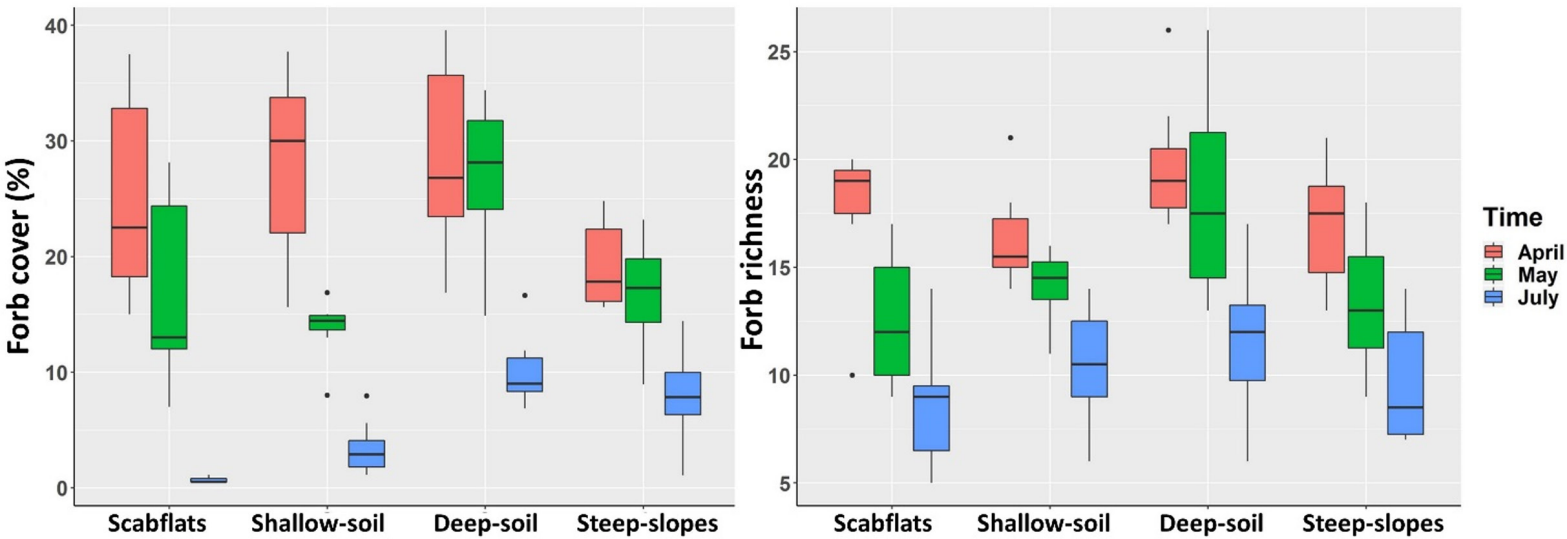
Boxplots of native perennial forb cover and richness stratified by vegetation group and sampling period

The cumulative average (combination of all three sampling periods) forb richness was 21.0 per plot; richness was highest in April (17.7), and lowest in July (10.2). Forb richness declined over time in all groups identified using cluster analysis (Figure 3; Appendix S1: Table A2 and S2). Forb richness was highest in both scabflats and deep‐soil sites in April (Figure 3). As the season progressed, a pattern of higher forb richness in deeper soils and the lowest richness in scabflats and steep slopes emerged (Figure 3). Forb mean cover (during spring peak) was about five times greater (25.6%) than mean July cover (5.5%, paired *t*‐test, *t* = 12.05, *p*‐value <.001, estimated difference = 20.1, 95% CI = 16.7 to 23.5). Forb cover was similarly high for scabflats, shallow soil, and deep soil sites and lowest for steep slopes in April. There was a pronounced shift in July to much greater forb cover in deep‐soil sites compared to the other groups (Figure 3). Forb cover decreased most dramatically from April to July in scabflats and the shallowest soils (Figure 3). The mean (plot‐level) ratio of forb peak cover to bunchgrass peak cover was 1.60 (quantiles: 0.25 = 0.77; 0.50 = 1.18; 0.75 = 2.04) compared to July where the mean ratio of forb cover to bunchgrass cover was only 0.41 (quantiles: 0.25 = 0.05; 0.50 = 0.20; 0.75 = 0.56).

**FIGURE 4:**
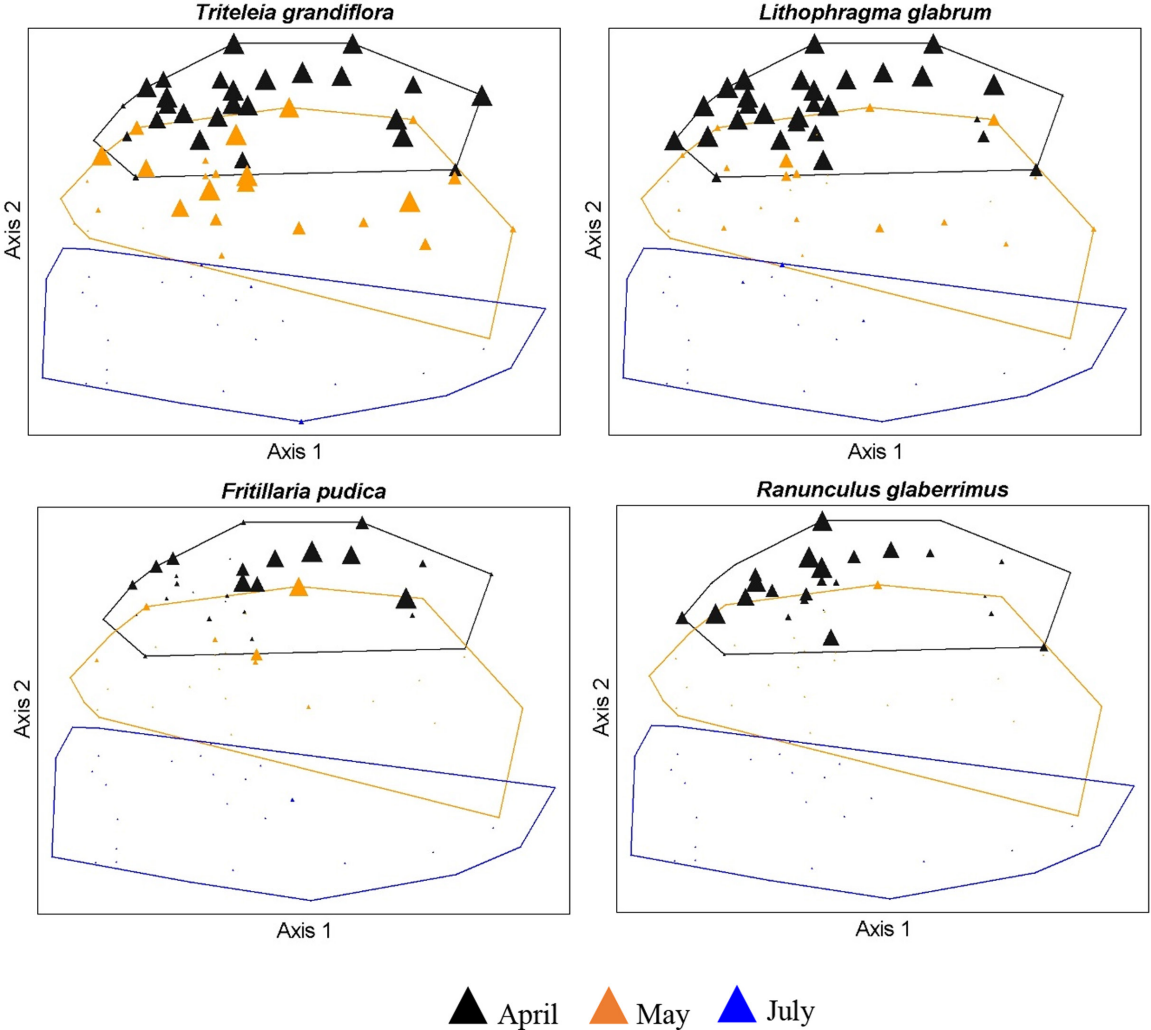
Selected species abundance in native perennial forb space for the three sampling periods. Symbol size corresponds to species abundance (% of quadrats occupied) for each plot. Color depicts sample period

Forb composition changed as a function of sampling period (*A* = 0.084, *p* < 0.001, MRBP). Pairwise comparisons revealed that forb composition differed between each sampling period. Compositional differences between April and May (*A* = 0.04, *p* < 0.001) and May and July (A = 0.04, p < 0.001) were relatively low; much greater community change occurred between April and July (*A* = 0.11, *p* < 0.001). Most of the variation (69%) in forb composition over the three time periods was explained by two ordination axes (Figure 4; Appendix S1: Figure A3). The strongest gradient (Axis 1; explaining 49% of variation in the distance matrix) was again associated with slope and soil P and K concentrations which all increased toward the right side of Axis 1 (Figure 4; Appendix S1: Figure A3). Axis 2 (explaining 20% of variation in the distance matrix) was associated with sampling period; April sample units were located at the top of the ordination space along Axis 2, May sample units were in the middle along Axis 2, and July sample units were clustered along the bottom of Axis 2 (Figure 4; Appendix S1: Figure A3). Most forb species (~70%) were positively correlated with Axis 2, and those with negative relationships, were relatively weak (*r* < −0.19), indicating tendencies for higher abundance of most forb species in spring that declined toward early summer. Forbs with the strongest associations toward earlier sampling periods tended to be the most ubiquitous perennial forbs including *T. grandiflora* (*r* = 0.79), *L. glabrum* (0.78), *F. pudica* (0.56), and *R. glaberrimus* (0.54; Appendix S1: Figure A3; Table 1). Species that were most associated with the later sampling periods were *Apocynum androsaemifolium* (−0.19), *Castilleja* spp. (−0.18), and *Scutellaria angustifolia* (−0.16). Consistent with NMS results, ISA indicated that most forb species showed tendencies toward either the April or May sampling periods compared to July (Table 1). ISA identified 17 significant (*p* ≤ 0.10) indicator species for the April–May time periods compared to only one indicator species (*Castilleja* spp.) for July (Table 1). The strongest indicator species for the early time periods were forbs that were among the most widespread across our study area including *T. grandiflora*, *L. glabrum*, *D. bicolor*, *H. pumilus*, and *Agoseris grandiflora* (Table 1). Several ubiquitous species, when all sampling periods were considered, *L. glabrum*, *T. grandiflora*, and *R. glaberrimus* that occurred in 84%, 77%, and 43% of quadrats, respectively, in April, were scarce or absent (occurring in 1%,1%, and 0% of quadrats, respectively) by July (Table 1; Figure 4). Along with *T. grandiflora* which experienced a factor of 67 decrease in abundance from April to July, other culturally important species, *F. pudica*, *L. cous*, and *C. quamash* abundances declined by a factor of 98, 7, and 2, respectively, from April to July (Figure 4; Table 1).

## 5 DISCUSSION

The majority of variation in forb composition (82%) was explained by two axes: (1) slope and soil P and K; and (2) soil depth. Axis 1 explained, by far, the most variation (66%) and represented a water drainage gradient. Plots at one end of this gradient were located on flat or convex sites (“scabflats”; Appendix S2) that were inundated with water in winter and spring because of a perched water table overlaying shallow impervious bedrock or clay (Daubenmire, 1942; Johnson & Swanson, 2005). At the other end of this gradient were steep slopes that facilitated water drainage. Soil depth was relatively consistent for sites at either end of Axis 1 indicating that restriction to rooting depth was not a major contributor to species variation along this gradient (but see Appendix S2). Scabflats experience the highest variability in soil hydrologic characteristics in our study area (rapid change from water inundation in spring to desiccation in early summer). Consistent with observations in temporarily flooded Mediterranean and tropical grasslands, geophytes (e.g., *C. quamash*, *L. bicolor*) were abundant (Keeley & Zedler, 1998, Esler et al., 1999, Gibson et al., 2005; relative abundance = 41%) in scabflats. High environmental variability is a strong driver of species distributions (Parepa et al., 2013). Extreme variation in hydrologic soil conditions imposes a special set of challenges to plants that are exposed to water inundation and desiccation during the growing season along with associated high variation in resource supply, geochemistry, and aeration (Parepa et al., 2013). Scabflat forbs may have higher tolerance for extreme growing season variation in soil hydrologic conditions and resource availability compared to forbs occurring higher along Axis 1 (with increased drainage). Alternatively, forb composition variation may be related to length of water inundation time. Duration of water inundation during the growing season is a strong driver of plant species distributions (Emery et al., 2019; Keeley & Zedler, 1998). Degree of water logging was the most important factor explaining variation in grass species in a European mesic grassland (Silvertown et al., 1999). Similarly, vegetation composition is strongly influenced by length of inundation time during the growing season in semi‐arid and arid grass and shrublands (Clausnitzer & Huddleston, 2002; Dippenaar, 2014; Gibson et al., 2005; Keeley & Zedler, 1998). Temporarily flooded soils (e.g., vernal pools or waterlogged soil at or near the surface) are ubiquitous in arid and semi‐arid regions with wet winters, dry summers, and perched water tables over impervious substratum such as in the interior Pacific Northwest, California, Australia, Chile, Middle East, Spain, and Africa (Clausnitzer & Huddleston, 2002). Researchers have identified the importance of temporary water inundation to support specialized vegetation communities as well as a need to better understand such patterns for conservation of biodiversity in arid and semi‐arid ecosystems (Clausnitzer & Huddleston, 2002; Dippenaar, 2014; Keeley & Zedler, 1998). Relationships between temporary flooding of arid and semi‐arid soils and plant community dynamics have been largely unexplored in Pacific Northwest steppe and bunchgrass ecosystems (Clausnitzer & Huddleston, 2002). Our finding that water drainage is the dominant gradient related to forb composition variation in our study area combined with a general lack of forb‐focused research in grasslands globally (Bråthen et al., 2021; Pokorny et al., 2004; Siebert & Dreber, 2019) suggests that successful management of diverse forb communities in the PNB and similar semi‐arid and arid grasslands with wet winters and summer drought requires increased understanding of interactions between soil hydrologic characteristics and forb community dynamics. Such knowledge is particularly important because stressors including land use, development, and climate change factors are expected to result in substantial alteration to soil hydrologic conditions in semi‐arid and arid regions (McMichael, 2004; Taylor et al., 2013). Our results suggest that alterations to soil hydrologic characteristics (i.e., drainage and timing of inundation) may have major impacts to native forb distributions in remnant PNB and similar grasslands.

The strong associations between Axis 1 and soil P and K coupled with our detection of soil nutrients as important predictors of abundance for many forb species, suggests that the availability of soil nutrients were also important for the delineation of forb species. Soil nutrients are often limiting resources for plant growth in natural grassland ecosystems and, therefore, are important factors affecting plant community composition and structure (Koerselman & Meuleman, 1996). Variations in soil nutrients alter plant species composition across different spatial scales, from large landscapes (Condit et al., 2013) to local sites spanning a few meters or less (Cavieres et al., 2008; Tilman, 1984). Effects of soil nutrient variation on plant species composition have been demonstrated across a wide range of terrestrial ecosystems for grasses, forbs, and shrubs/trees (Cavieres et al., 2008; Condit et al., 2013; Tilman, 1984). For example, fine‐scale effects of natural variation in soil K concentration have been documented to influence forb (*Taraxacum officinale*) abundance, fitness, and survival between soil under nurse plants compared to adjacent interspaces in alpine habitats in Chile (Cavieres et al., 2008). We found that *C. quamash*, *L. bicolor*, *O. douglasii*, and *H. pumilus* were the forbs most strongly associated with lower P and K availability and that *E. heracleoides*, *A. reventus*, *L macrocarpum*, and *L. cous* were strongly associated with higher P and K. We also found that soil nutrients explained much more variation in abundance for some forbs compared to the ordination space. For example, the ordination axes explained <1% and 19% of the variation in *G. nana* and *F. pudica* abundance, respectively. Whereas, 75% of *G. nana* variation was explained by CEC, Elevation and K, and 42% of *F. pudica* variation was explained by Mg, K, and Ca. Unfortunately, little is known about how PNB forb composition relates to variation in soil nutrients. Soil characteristics and descriptions have long been used to describe variations in PNB bunchgrass composition (Daubenmire, 1942; Johnson & Simon, 1987), and are important predictors of native and non‐native species abundance in the PNB (Hanson et al., 2008). However, the contribution of soil nutrients to species composition gradients has not been separated out from other soil covariates such as water availability resulting in a major knowledge gap regarding the influence of soil nutrient variation on perennial forb composition gradients and specific forb distributions/performance in the PNB. Future research will be needed to separate the effects of nutrient availability from other edaphic characteristics on PNB forb community variation.

Because the PNB is a semi‐arid grassland, it may be expected that growing season water availability would be the most important factor influencing plant species composition (Burke et al., 1998). However, water availability was the secondary gradient, explaining 16% of forb composition variation. Rhizomatous and tap‐rooted forbs (*Arnica sororia*, *P. gracilis*, *P. glandulosa*, and *A. millefolium*) had high affinities to the mesic sites. Our findings were consistent with previous description of mesic PNB bunchgrass sites where rhizomatous forbs and those that peak during the summer phenological optimum of bunchgrasses are primarily found in mesic bunchgrass communities associated with *F. idahoensis* and *K. macrantha* (Daubenmire, 1942, Johnson & Swanson, 2005, Appendix S2). At the other end of the soil depth gradient corresponding to the most xeric sites, geophyte relative abundance was highest and rhizomatous relative abundance was lowest. Forbs with the strongest affinities to shallow soils were spring ephemerals that senesce by early summer (e.g., *A. acuminatum*, *A. tolmiei*, *L. cous*, and *R. glaberrimus)*. The more extreme summer drought conditions in the shallowest soil sites compared to deeper soils likely explains the dominance of spring ephemeral geophytes in shallower soils. Summer‐dormant geophytes are specially adapted to short growing seasons in the spring when water is available followed by summer dormancy to escape drought (Batanouny, 2001); geophyte abundance is generally higher in habitats with high variation in resource availability and short growing seasons such as in Mediterranean, steppe, and shallow soil habitats (Batanouny, 2001; Esler et al., 1999; Vymazalova et al., 2016). Previous research in the PNB suggested that highly preferred geophytes (by fossorial animals, e.g., *A. tolmiei*, *L. cous*) may be restricted to stony sites where they are inaccessible to underground herbivory by small mammals (Cox, 1989). Fossorial herbivores can have profound effects on vegetation distributions. For example, pocket gophers (*Thomomys bottae*) limited aspen stands to rock outcrops where they were protected from herbivory in mountain meadows in Arizona (Cantor & Whitham, 1989). Fossorial herbivores can have both positive and negative effects on forb abundance depending on herbivore and grassland characteristics (Andersen, 1987; Davidson et al., 2012). Future research will be needed to determine the role of below ground herbivory versus water availability to forb species establishment along soil depth gradients in the PNB.

Our results demonstrate that native perennial forb composition, richness, and abundance changed substantially from spring to early summer. The vast majority of forbs had affinities to spring and half of the total forb species were not even observable during the summer period, which coincides with most vegetation monitoring efforts in the region (Endress et al., 2022). Our results suggest that vegetation sampling coinciding with the summer phenological optimum will grossly underrepresent forb abundance and distributions in the PNB. Data gaps will be particularly severe for forb assemblages that occur on flat, poorly drained sites overlaying shallow perched water tables and the shallowest, most drought‐prone habitat. Plant community composition and diversity often differs considerably between spring and summer plant assemblages (Pokorny et al., 2004; Ristau & Horsley, 2001; Tremlay & Larocque, 2001; Vymazalova et al., 2012). Large scale vegetation sampling efforts are often biased toward the summer phenological optimum (Endress et al., 2022; Pokorny et al., 2004; Vymazalova et al., 2012), which occurs after many grassland forbs have senesced (Endress et al., 2022; Pokorny et al., 2004). Additionally, forbs have been a secondary focus in grasslands (Bråthen et al., 2021; Pokorny et al., 2004; Siebert & Dreber, 2019). The paucity of forb‐focused research and monitoring in grasslands coupled with timing of vegetation sampling that is out of phase with forb phenology, suggests major gaps in our understanding of forb community dynamics and distributions in grasslands (Endress et al., 2022; Pokorny et al., 2004; Siebert & Dreber, 2019). For example, previous descriptions of PNB plant communities include increased dominance of forbs with more mesic conditions. More specifically, the greatest bunchgrass dominance and lowest representation of perennial forbs is expected in scablands and on sites with the shallowest, most droughty, soils (dominated by *P. secunda*, *D. unispicata*, and *P. spicata*). In contrast, the highest representation of perennial forbs is expected in the most mesic, *F. idahoensis‐*dominated communities (Daubenmire, 1942; Johnson & Swanson, 2005). Our results do not support these descriptions, and suggest that previous understanding of PNB community structure has focused on the summer phenological optimum of bunchgrasses and are, therefore, incomplete. We found that perennial forb cover, in April, was comparable between scabflats (dominated by *D. unispicata* and *P. secunda*), shallow soil (*P. secunda*), and deep soil sites (*F. idahoensis*, *K. macrantha*). Species richness was also comparable between the scabland and deeper soil sites during April. The previously held assumption of higher abundance of forbs in more mesic sites only held true when spring data were ignored. We found that peak perennial forb cover in spring was about as high, and in many sites, greater than peak bunchgrass cover across our study area. This finding combined with belowground biomass estimates of dominant PNB geophytes (i.e., *C. quamash*, and *L. cous*) accounting for ~80% of their total biomass during peak aboveground production (Averett and Endress unpublished data; signifying that forbs are even more abundant in terms of belowground biomass compared to what above ground observations may suggest), and that the contribution of annual forbs, which are abundant in the PNB but were not considered in this study, underscore the importance of forbs in the PNB and a need to understand forb community dynamics and relationships to grassland processes and function in the PNB and similar grasslands.

## 6 CONCLUSION

Conservation of native forbs is important for ecological and social functions/services in grasslands. However, there is little ecological information to inform management of such species in many grasslands. This is the only peer‐reviewed study, to our knowledge, to take a forb‐centered approach for evaluating vegetation community gradients and intra‐annual variation in a PNB grassland. Our results demonstrate that most of the variation in forb community composition was explained by water drainage and secondarily by soil depth. The high importance of water drainage to variation in forb composition suggests a need to understand how soil hydrologic characteristics such as timing of water inundation and drought relate to grassland forb communities and how expected changes to soil hydrologic conditions in the PNB and similar grasslands may impact grassland forbs. Our study was limited to one grassland system in eastern Oregon. Future research will be needed to determine relationships between forb communities, hydrology, slope, and edaphic factors across the full spectrum of PNB grasslands as well as extending research to similar grasslands in other regions. Our results show that summer sampling, coincident with peak grass production, is inappropriate for sampling forb communities in the PNB; such sampling will grossly underrepresent forb abundance, richness, and distributions. These errors will be particularly severe for poorly drained sites overlaying shallow perched water tables (in spring) and forbs inhabiting the shallowest most drought‐prone soils. General biases toward summer sampling and grass‐focused research coupled with similar patterns of abundant summer‐dormant forbs in semi‐arid and arid grasslands with comparable precipitation patterns and soil hydrologic characteristics suggest that forb communities may be underrepresented in other grasslands as well. Future research is needed to better understand how timing of conventional vegetation sampling may influence our understanding of forb community dynamics in grasslands globally.

## AUTHOR CONTRIBUTIONS

**Joshua Averett:** Conceptualization (supporting); data curation (lead); formal analysis (lead); investigation (equal); methodology (equal); validation (equal); visualization (lead); writing – original draft (lead); writing – review and editing (equal). **Bryan Endress:** Conceptualization (lead); data curation (supporting); formal analysis (supporting); funding acquisition (lead); investigation (equal); methodology (equal); project administration (lead); supervision (lead); validation (equal); visualization (supporting); writing – original draft (supporting); writing – review and editing (equal).

## CONFLICT OF INTEREST

The authors declare that they have no conflict of interests.

## Supporting information


Appendix S1‐S2

File S1

## Data Availability

All data needed to reproduce our results are available on Data Dryad at https://doi.org/10.5061/dryad.xksn02vj9.
