# Supplementary material for: Forb composition gradients and intra‐annual variation in a threatened Pacific Northwest Bunchgrass Prairie
Source: Ecol Evol. 2022 Jun 22;12(6):e9021. doi: 10.1002/ece3.9021 (PMC9217882; doi:10.1002/ece3.9021)
Supplement: Supplementary file 1 — Appendix S1‐S2 [file ECE3-12-e9021-s001.docx]

Appendix S1: Supplementary Figures and Tables

N

0 m

r = 7 m

1 m

1.75 m

4.5 m

2.0 m

3.5 m

3.5 m

4.5 m

W

E

7 m

6.75 m

9.5 m

10.5 m

12.25 m

12 m

13 m

14 m

S

Figure A1: Circular vegetation sampling plot. Shaded squares indicate placement of (1 m2) vegetation quadrats. Shaded circles are soil depth measurement locations; open points are locations of soil samples. Solid lines show the North/South and West/East transect lines; hashed lines are the North West/South East and North East/South West transect lines


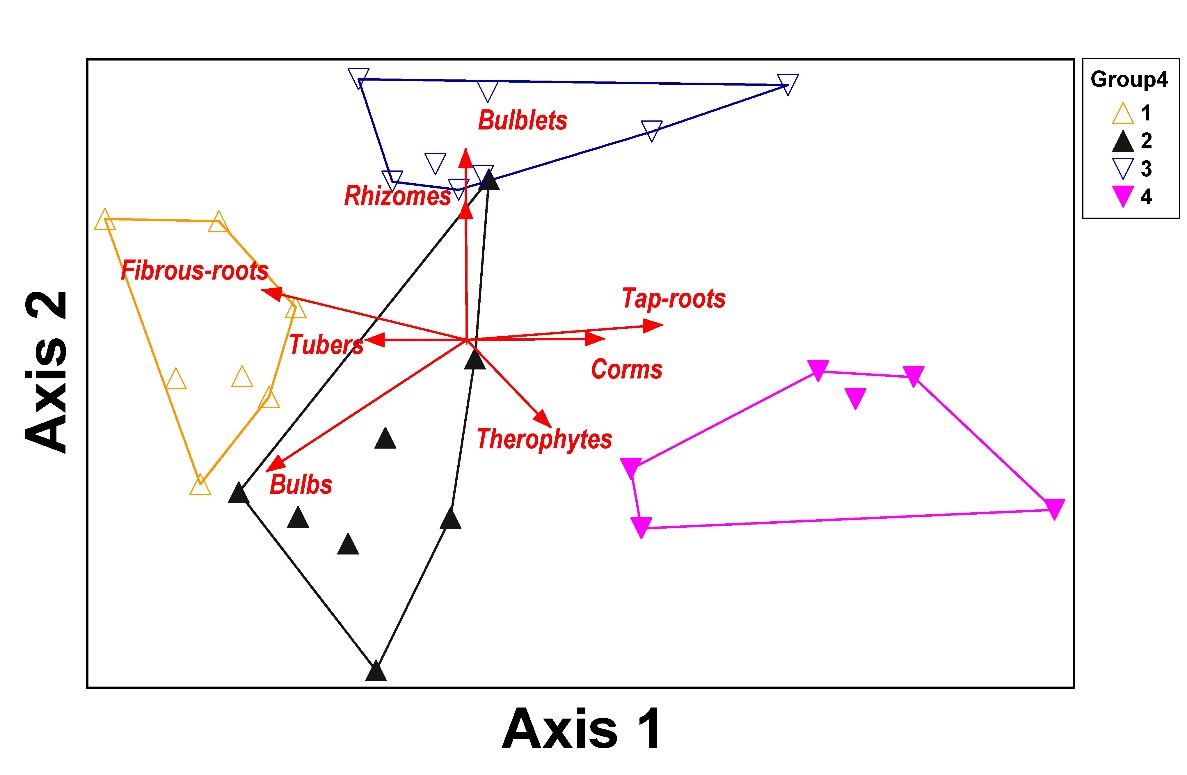


Figure A2: NMS ordination of sample units in native perennial forb species space. Axis 1 and Axis two represent 66% and 16% of variation in the distance matrix respectively. Vectors show direction and strength (length) of linear correlations between relative abundance of forbs with specific root/below ground storage structures and the ordination space. Therophytes are annual forbs.

April

May

July


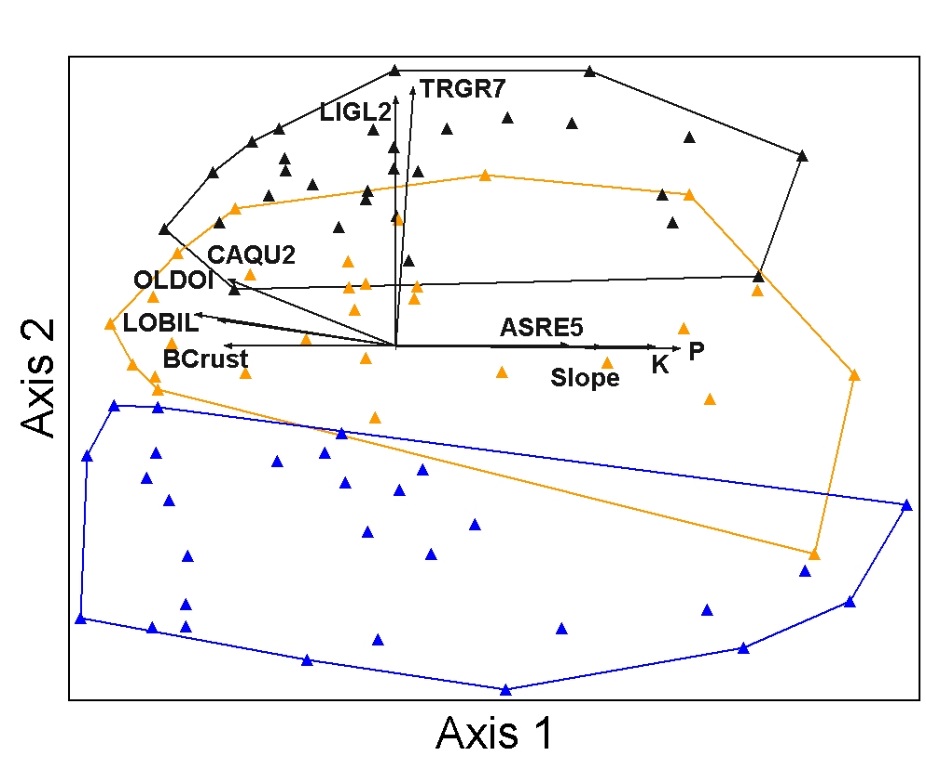


Figure A3: NMS ordination of plots (triangles) in native perennial forb space for the three sampling periods represented by symbol and convex hull color. Axis 1 (explaining 49% of variation in the distance matrix) and axis 2 (explaining 20%) show the dominant community gradients. Linear relationships between the strongest related environmental and species responses and the ordination space are represented by vectors.

Table A1: Representation (mean proportion) of native perennial forbs with selected root/underground storage morphology by vegetation group. Standard deviations are shown parenthetically. Geophytes included species with large belowground storage structures, i.e., bulbs, corms, tubers, belowground bulblets and/or cormlets.

| **Vegetation Group** | **Fibrous-rooted** | **Geophyte** | **Rhizomatous** | **Tap-rooted** |
| --- | --- | --- | --- | --- |
| **Group 1** (Scablands) | 0.26 (0.06) | 0.41 (0.05) | 0.13 (0.03) | 0.20 (0.06) |
| **Group 2** (Shallow soil) | 0.24 (0.05) | 0.49 (0.06) | 0.09 (0.03) | 0.18 (0.05) |
| **Group 3** (Deep soil) | 0.21 (0.04) | 0.40 (0.05) | 0.16 (0.04) | 0.22 (0.08) |
| **Group 4** (Steep slopes) | 0.17 (0.03) | 0.35 (0.13) | 0.13 (0.10) | 0.34 (0.11) |

Table A2: Indicator species analysis results showing native perennial forbs with strong affinities towards each of the four groups described using cluster analysis. Plot level relative abundance and relative frequency, indicator value, and p-values are shown for each species. Only species with (IV) p-values < 0.05 are included in this table.

| **Species** | **Rel.Abund (%)** | **Rel.Freq (%)** | **IV** | **p-value** |  |
| --- | --- | --- | --- | --- | --- |
| **Group 1 (n = 7)** | | | | | |
| *Hesperochiron pumilus*  *Idahoa scapigera*  *Orobanche uniflora*  *Polygonum polygaloides* | 43 | 100 | 42.7 | < 0.01 |  |
| *Balsamorhiza serrata* | 47 | 86 | 40.7 | 0.03 |  |
| *Camassia quamash* | 38 | 100 | 37.5 | < 0.01 |  |
| *Lomatium bicolor* | 36 | 100 | 35.6 | < 0.01 |  |
| *Balsamorhiza incana* | 50 | 71 | 35.6 | 0.05 |  |
| *Trifolium macrocephalum* | 59 | 57 | 33.7 | 0.05 |  |
| *Olsynium douglasii* | 31 | 100 | 31.1 | < 0.01 |  |
| **Group 2 (n= 8)** | | | | | |
| *Allium fibrillum* | 64 | 75 | 47.8 | 0.01 |  |
| *Allium tolmiei* | 58 | 75 | 43.7 | 0.01 |  |
| *Ranunculus glaberrimus* | 38 | 100 | 37.6 | < 0.01 |  |
| **Group 3 (n = 8)** | | | | | |
| *Perideridia gairdneri* | 73 | 100 | 73.3 | <0.01 |  |
| *Sidalcea oregana* | 90 | 75 | 67.6 | < 0.01 |  |
| *Arnica sororia* | 88 | 75 | 66.2 | < 0.01 |  |
| *Saxifraga nidifica* | 60 | 88 | 52.5 | < 0.01 |  |
| *Potentilla gracilis* | 100 | 50 | 50.0 | 0.01 |  |
| *Lithophragma parviflorum* | 55 | 88 | 47.8 | < 0.01 |  |
| *Achillea millefolium* | 44 | 100 | 43.8 | < 0.01 |  |
| *Potentilla glandulosa* | 100 | 38 | 37.5 | 0.04 |  |
| **Group 4 (n = 6)** | | | | | |
| *Astragalus reventus* | 100 | 83 | 83.3 | < 0.01 |  |
| *Lomatium macrocarpum* | 81 | 67 | 54.3 | < 0.01 |  |
| *Eriogonum heracleoides*  *Bromus spp.*  *Poa pratensis*  *Festuca idahoensis* | 50 | 83 | 41.8 | 0.02 |  |
| *Crepis spp.* | 100 | 33 | 39.4 | 0.04 |  |
| *Lomatium cous* | 56 | 67 | 37.4 | 0.03 |  |
| *Galium boreale* | 37 | 100 | 33.3 | 0.03 |  |

Appendix S2: Cluster analysis, plant community group descriptions, and relationships to the dominant non-metric multidimensional scaling ordination axes.

**Cluster analysis**

The cluster dendrogram was pruned at four groups with ~ 36% of information remaining in the distance matrix. The four community groups separated clearly in NMS ordination space (Averett and Endress In Review; Fig. A2). **Group 1** was located to the far-left along Axis 1. This group consisted of plots with the highest elevations, flattest topography, shallow to moderate soil depths, and the lowest soil pH, P, and K concentrations (Fig. 2; Table 2). These sites are referred to regionally as ‘scablands’ or ‘scabflats’ (Daubenmire 1942; Johnson and Swanson 2005). NPF species with the strongest affinities to group one included *Hesperochiron pumilus*, *B. serrata*, *C. quamash*, *L. bicolor*, and *Balsamorhiza incana* (Table 3). *Danthonia unispicata* was the native bunchgrass most associated with Group 1 (Fig. 2). Fibrous rooted forbs (relative abundance = 26%) and geophytes (41%) were highly represented in Group 1 (Fig. 2). Geophytes with bulbs were particularly well represented within Group 1 (Fig A2).

**Group 2** consisted of plots at moderate to high elevation, with gentle to moderate slopes, greater cover of rock and bare ground, with extremely shallow soil depth (Fig. 2; Table 3). Consistent with Group 1, plots in Group 2 were located in areas with comparatively low soil pH, P, and K (Fig 2; Table 3). *A. fibrillum, A. tolmiei,* and *Ranunculus glaberrimus* were NPF species with strong affinities to Group 2 (Fig 2; Table 3). *Poa secunda* was the native bunchgrass most associated with Group 2 (Fig. 2). Group 2 had the highest representation of geophytes (relative abundance = 49%) and the lowest representation of rhizomatous (9%) and tap-rooted (18%) forbs (Fig. 2; Table A1).

**Group 3** was located mid-way along Axis 1 and at the top of Axis 2 corresponding to gentle slopes with the deepest soils in our study area (Fig 2; Table 2). Plots in group 3 also contained the highest percent clay and greater P and K compared to groups 1 and 2 (Fig. 2; Table 2). *Perideridia gairdneri*, *Sidalcea oregana*, *Arnica sororia*, *S. nidifica*, *Potentilla gracilis*, *L. parviflorum*, *A. millefolium*, and *Potentilla glandulosa* were NPF indicators for Group 3 (Table 3). *Koeleria macrantha*, and *F. idahoensis* were the bunchgrasses most associated with Group 3 (Fig. 2). Group 3 had the highest representation of rhizomatous forbs (relative abundance = 16%) in our study area (Fig. 2; Table A1).

**Group 4** was located to the far right along Axis 1 and low along Axis 2 corresponding to sites with much steeper slopes, higher soil pH, P, and K concentrations, and shallow to moderate soil depth (Fig 2; Table 2). Species with strong affinities for Group 4 included *A. reventus*, *Lomatium macrocarpum*, *E. heracleoides*, *Crepis spp*., *Lomatium cous*, and *Galium boreale* (Table 3). *Pseudoroegneria spicata* was the native bunchgrass with the strongest affinity to Group 4 (Fig. 2). Group 4 had the highest representation of tap-rooted forbs (relative abundance = 34%; Fig. 2; File A1).

The primary gradient in NPF composition was related to soil nutrients (P and K) and slope. We suspect that the dominant factor driving separation of forb species along Axis 1 was related to water drainage and more specifically plant species tolerance to waterlogging. Plots at one end of this gradient were located on flat sites that restricted water drainage (scabflats). Several summer-dormant geophytes (e.g., *C. quamash*, *L. bicolor*, *H. pumilus*) were strongly associated with these plots. *Camassia quamash* occurs in seasonally wet habitats in the Pacific Northwest that are inundated with water in the spring and dry out by late-spring or early-summer (Beckwith 2004). Similarly, *L. bicolor* has been described as being affiliated with perched water tables in the spring (Johnson and Swanson 2005) or heavy clay soils (Hitchcock et al. 1955) that have poor drainage. Vegetation Group 1 was most associated with this end of the gradient, and closely aligns with the *D. unispicata*/*L. bicolor* plant association (Johnson and Swanson 2005). This plant association occurs on sites locally referred to as “scabflats” (Daubenmire 1942; Johnson and Swanson 2005) that are characterized by flat or convex topography with water saturated soil in spring due to a perched water table overlaying a shallow impervious layer of bedrock and/or clay (Daubenmire 1942; Johnson and Swanson 2005). Scablands experience the strongest contrasting soil conditions (rapid change from water inundation to desiccation during spring and frequent frost heaving in winter) compared to other soils in the PNB (Daubenmire 1942). Species experiencing such conditions require special adaptations that allow them to grow during periods of water inundation (Crawford 1996) as well as survive prolonged dehydration (Volaire and Norton 2006). Perennial forbs with bulbs were particularly well represented within scablands and shallow soil sites and reflected the high abundance of *C. quamash* and *Allium* species found in Groups 1 and 2 (Figs. 2 and A2). At the other end of this gradient, were plots (Group 4) found on the steepest slopes which facilitated water drainage. Forbs most associated with steep terrain included strongly tap-rooted species, *A. reventus* and *E. heracleoides* that maintain growth throughout the summer. Geophyte relative abundance was lowest in the steepest terrain. It is important to note, that while we found a negative relationship between geophyte relative abundance and NMS Axis 1, geophytes were still the most highly represented forb life-form in all vegetation groups (Table A1), underscoring geophyte importance in the PNB. Widely distributed geophytes (i.e., *T. grandiflora*, *L. glabrum*, *D. bicolor*) helped maintain the high representation of geophytes (35-49% of forb cover) across our entire study area (Fig. 1; Table A1). Since species at both ends of this gradient experience drought conditions during summer, we expect that the degree to which species are adapted to temporary water inundation was more important for separation of species along Axis 1 compared to adaptations related to drought tolerance.

Because the PNB is a semiarid grassland, we may expect the primary driver of plant community structure to be growing season water availability (Burke 1998). However, our results suggest that variations in water drainage and/or soil nutrients explained more vegetation community variation compared to season long water availability (i.e., drought tolerance; represented along Axis 2). These findings are consistent with previous research that reported that species tolerance to waterlogging can explain more variation in grassland community composition compared to drought tolerance and is a strong force in structuring grassland composition and promoting niche segregation in such habitats (Silvertown et al. 1999). We found that soil depth and texture (both related to water holding capacity) were strongly related to Axis 2, which is independent from Axis 1. Therefore, most of the composition variation due to season-long water availability (or drought tolerance) should be captured along Axis 2. However, some evidence suggests that species patterns along Axis 1 may be at least partially related to differences in restrictions to rooting depth. Access to water deeper in the soil profile extends water availability later into the growing season. Differences in soil depth existed between Group 4 (19.1 cm) and Group 1 (14.4 cm), the extremes along Axis 1. However, all of the soil depths measured were relatively shallow and consistent with scabland soil depths reported (< 30 cm) in the region (Johnson and Simon 1987; Johnson and Swanson 2005). *Eriogonum heracleoides* and *P. spicata* have been described as requiring deeper soil than those supported by scablands for establishment in the region (Weaver 1915; Johnson and Simon 1987). The strong associations of *E. heracleoides* and *P. spicata* with Group 4 suggest that the actual rooting depths may be deeper than the soil depths measured at those sites. We suspect that the discrepancy between our detection of species requiring deeper soil than we measured on the steepest slopes was due to either a high presence of large stone fragments and/or fractured basalt shallow in the soil profile which allowed for deep root penetration between rocks or along bedrock fissures, but limited soil probe penetration for soil depth measurements.

The secondary gradient in NPF composition corresponded to a soil depth gradient. Plots at the top of this gradient (Fig. 2) were found on the deepest soils in our study area. Native perennial forbs with the strongest associations with deeper soils were two geophytes, *S. nidifica* and *P.gairdneri*. Group 3 was clustered at the top end of the soil depth gradient corresponding to the deepest soils. Species with strong affinities to Group 3 included tap-rooted, and many rhizomatous forbs, *S.*, *Arnica sororia*, *P. gracilis*, *P. glandulosa* and *A. millefolium,* characteristic of mesic PNB habitats. Clay content was also highest in the deeper soil sites. Deeper soil combined with finer soil texture indicates greater water holding capacity for plant use later in the growing season compared to the shallower soils with coarser texture found low along Axis 2. The deeper soil plots aligned more closely with the *F. idahoensis* and *P. spicata/F. idahoensis* series described by Johnson and Simon (1987) and Johnson and Swanson (2005). These plant associations occur over increasingly more mesic soils (with higher dominance of *F. idahoensis*) compared to those dominated by *P. spicata* or scabland bunchgrasses (*P. secunda* and *D. unispicata*). At the bottom of the soil depth gradient were plots on extremely shallow (average ~ 10 cm; 4 in) soils with relatively high cover of rock (9%), bare ground (23%), and dominance of *P. secunda*, the most drought tolerant of the PNB bunchgrasses. Forbs with strong affinities for these sites were *A. acuminatum*, *A. tolmiei* , and *R. glaberrimus*. Previous researchers have hypothesized that highly preferred geophytes (by fossorial animals, e.g., *A. tolmiei*, *L. cous*) may be restricted to stony sites where they are inaccessible to underground herbivory by small mammals (Cox 1989). Future research will be needed to determine the role of herbivory versus environmental limitations to species establishment along soil depth and, soil physical and chemical gradients in the PNB.

Beckwith, BR. 2008. The queen root of this clime: ethnoecological investigations of blue camas (Camassia leichtlinii (Baker) Wats., C. quamash (Pursh) Greene; Liliaceae) and its landscapes on southern Vancouver Island, British Columbia. Dissertation. University of Victoria.

Crawford RMM. 1996. Whole plant adaptations to fluctuating water tables. Folia Geobotanica and Phytotaxonomica, 31: 7-24.

Hitchcock CL, Cronquist A. 1955. Flora of the Pacific Northwest. University of Washington Press. Seattle and London.

Volaire F, Norton M. 2006. Summer dormancy in perennial temperate grasses. Annals of Botany, 98: 927-933.
